# Telling the Seasons Underground: The Circadian Clock and Ambient Temperature Shape Light Exposure and Photoperiodism in a Subterranean Rodent

**DOI:** 10.3389/fphys.2021.738471

**Published:** 2021-10-01

**Authors:** Danilo E. F. L. Flôres, Milene G. Jannetti, Giovane C. Improta, Patricia Tachinardi, Veronica S. Valentinuzzi, Gisele A. Oda

**Affiliations:** ^1^Laboratorio de Cronobiologia Binacional Argentina-Brasil, Departamento de Fisiologia, Instituto de Biociências, Universidade de São Paulo, São Paulo, Brazil; ^2^Laboratorio de Cronobiologia Binacional Argentina-Brasil, Centro Regional de Investigaciones Cientificas y de Transferencia Tecnológica (CRILAR), Anillaco, Argentina

**Keywords:** photoperiod, subterranean rodent, extreme photic environment, light exposure, circadian clock, biologging, activity patterns, mathematical modeling

## Abstract

Living organisms anticipate the seasons by tracking the proportion of light and darkness hours within a day—photoperiod. The limits of photoperiod measurement can be investigated in the subterranean rodents tuco-tucos (*Ctenomys* aff. *knighti*), which inhabit dark underground tunnels. Their exposure to light is sporadic and, remarkably, results from their own behavior of surface emergence. Thus, we investigated the endogenous and exogenous regulation of this behavior and its consequences to photoperiod measurement. In the field, animals carrying biologgers displayed seasonal patterns of daily surface emergence, exogenously modulated by temperature. In the laboratory, experiments with constant lighting conditions revealed the endogenous regulation of seasonal activity by the circadian clock, which has a multi-oscillatory structure. Finally, mathematical modeling corroborated that tuco-tuco’s light exposure across the seasons is sufficient for photoperiod encoding. Together, our results elucidate the interrelationship between the circadian clock and temperature in shaping seasonal light exposure patterns that convey photoperiod information in an extreme photic environment.

## Introduction

Temporal organization of physiology and behavior is achieved by the coordination of biological rhythms at all-time scales. Among these are the nearly 24 h circadian rhythms, which are synchronized by the daily alternation between light (L) and darkness (D) in the 24 h light/dark (LD) cycle (Aschoff, 1981). Throughout the year, the day length varies predictably, from short-days in winter to long-days in summer, generating annual changes in photoperiod, i.e., the proportion between L and D hours within a day. Accordingly, many organisms use photoperiod as an anticipatory cue to synchronize seasonal rhythms such as reproduction, hibernation and migration (Bradshaw and Holzapfel, 2007; Hut et al., 2013). “Extreme photic” environments, for instance, the subterranean, caves, poles and the deep sea are natural contexts that provide insights into the persistence of biological rhythms, as well as their synchronization by light/dark cycles (Lu et al., 2010; Duboué and Borowsky, 2012; Williams et al., 2014, 2016; Beale et al., 2016). Particularly, organisms that inhabit atypical photic environments have revealed striking new aspects of seasonal regulation by light signals (Appenroth et al., 2021; Rajan et al., 2021).

South American desert subterranean rodents known as the Anillaco tuco-tucos (*Ctenomys* aff. *knighti*) emerge to the surface several times a day (Tomotani et al., 2012). We have previously shown, under controlled laboratory conditions, that tuco-tucos display robust circadian rhythms in physiology and behavior (Valentinuzzi et al., 2009; Tachinardi et al., 2014, 2015). Moreover, our visual observations in field enclosures revealed irregular daily patterns of light exposure (Tomotani et al., 2012) which were later confirmed by light-sensing biologgers attached to individual animals (Flôres et al., 2016). Nevertheless, our computational studies, along with experimental tests, indicated that this irregular light exposure was sufficient for the day-night synchronization of their circadian rhythms (Flôres et al., 2013, 2016). Here we extend this investigation to the annual, seasonal synchronization by varying photoperiods.

Photoperiodic time measurement has usually been studied in the laboratory, by exposing animals continuously to (complete) short- or long-day photoperiods, under artificially imposed LD cycles (DeCoursey, 1972; Elliott, 1976). In contrast, tuco-tucos in nature expose themselves to light only when they actively emerge from their dark burrows. They emerge daily to forage, watch the surroundings, or perform tunnel maintenance (Tomotani et al., 2012). Consequently, they are exposed to self-imposed light regimens, similar to other burrow-dwelling rodents (DeCoursey, 1986; Pratt and Goldman, 1986; Williams et al., 2017). Thus, we hypothesized that tuco-tucos could experience different daylengths throughout the year if they modified their timing of surface emergences across seasons. For instance, they could emerge earlier and/or retreat later each day in summer as compared to winter, thus getting a cue of photoperiod variation from the self-imposed light regimen. Here, we thus investigated the exogenous and endogenous factors that determine when during the day tuco-tucos leave their tunnels and see the light. Seasonal changes in these factors are likely to shape the temporal pattern of light exposure at different seasons, with consequences to photoperiod measurement.

Several field studies have underscored the role of temperature as an important exogenous factor shaping daily activity patterns in small and diurnal desert rodents across the seasons. While in winter the incidence of active animals is higher around noon, it often becomes bimodal in summer, concentrated in twilight times. These seasonal changes in activity patterns are interpreted as a response of epigeous rodents to the daily variations in ambient temperature: in winter, small rodents concentrate foraging activity in warmer midday hours, whereas in summer, they avoid high midday heat loads by retreating under shade spots or burrow entrances (Kenagy et al., 2002; Bacigalupe et al., 2003). Since tuco-tucos expose themselves frequently to the surface (Flôres et al., 2016), we hypothesize that ambient temperature may also be a strong exogenous factor modulating their surface emergences.

The endogenous factor regulating emergence time is visible through the seasonal change in the daily activity duration (interval between activity onset and offset within a day) (Daan and Aschoff, 1975; Kenagy, 1976; Halle, 1995). Chronobiology studies have long investigated the role of photoperiod alone in changing activity patterns through laboratory experiments under constant temperature. In mammals, endogenous circadian rhythms in physiology and behavior are coordinated by a circadian clock in the suprachiasmatic nuclei (SCN), which is entrained (synchronized) by the daily LD cycle via direct input from the retina (Tackenberg and McMahon, 2018). The SCN dictate the timing of gross activity and rest, thus shaping the 24 h activity pattern. When photoperiod is artificially manipulated in laboratory experiments, the SCN-regulated activity/rest rhythm displays a notorious change in the duration of daily activity, replicating the changes observed across seasons in nature. We herein use the term “α” from the circadian literature to describe this daily activity interval duration, when it is measured in the laboratory (Pittendrigh and Daan, 1976a, b, c; Tackenberg et al., 2020). Under artificial long days, α is longer or shorter for light-active or dark-active organisms, respectively. Further, it has been shown that the changes in α are paralleled in the SCN electrical activity rhythm (Houben et al., 2009), that is, photoperiod-dependent α can be explained by different SCN entrainment patterns. A model was proposed by Pittendrigh and Daan (1976c) to explain these α changes, in which the circadian clock is composed of two coupled oscillators, each tracking dawn or dusk. One evidence for this proposition is the “splitting” of activity into two daily bouts which is observed in rodents maintained under artificial constant light (LL) for several days. The splitting phenomenon is considered the hallmark of the dual structure of the clock (Pittendrigh and Daan, 1976c; Oda and Friesen, 2002; Rohling and Meylahn, 2020).

In tuco-tucos, we predict an unusual bidirectional relationship between endogenous timing and exposure to light under different photoperiods: the endogenous regulation of daily activity rhythms may contribute to the seasonal changes in surface activity and light exposure patterns; conversely, the resulting light/dark patterns are predicted to entrain the circadian clock and feedback on activity control.

In the present work, we integrate ecophysiological and chronobiological approaches to investigate seasonal regulation in the timing of surface emergences during light hours and its consequences to light exposure and photoperiod encoding in tuco-tucos. First, we investigated the seasonal differences in light exposure and field activity onset/offset times from animals in field enclosures, using miniature biologgers. Ambient temperature was evaluated as an exogenous factor affecting the timing of surface emergences during the day. Secondly, we examined the endogenous rhythmicity of animals captured during winter and summer and released directly into laboratory constant darkness (DD). Persistent, historic-dependent rhythmic patterns—“aftereffects”—are expected in circadian oscillators entrained to different photoperiods (Tackenberg et al., 2020). Third, we investigated the multi-oscillatory nature of the circadian clock of tuco-tucos, by testing the occurrence of the “splitting” phenomenon under prolonged LL. Finally, we developed a mathematical model to test minimum light inputs for photoperiod encoding, mimicking our field data. Together, our results elucidate the interrelationship between the circadian clock and temperature in shaping seasonal light exposure patterns that convey photoperiod information in this extreme photic environment, contributing to the knowledge built from traditional laboratory experiments.

## Materials and Methods

### Field Study Area

This study was conducted in the facilities of CRILAR (*Centro Regional de Investigaciones Científicas y de Transferencia Tecnológica*), located in Anillaco, La Rioja, Argentina (28°48′S; 66° 56′ W; altitude: 1,350 m). The study area belongs to the northern region of the Monte Desert. At the summer solstice, the duration of the photophase (the “L” portion in an LD cycle) in this area is approximately 14 h, being 3 h and 40 min longer than the photophase during the winter solstice (approx. 10 h and 20 min) (Time and Date AS, 2021). The population of tuco-tucos studied (*Ctenomys* aff. *knighti*) occurs naturally in this area.

### Animal Capture and Husbandry

Animals were trapped using tubular traps made of rigid plastic (PVC), placed in the entrance of active burrows. The traps did not injure or offer any threat to animals’ bodily integrity and, to minimize any discomfort and stress, each trap was checked at least every 2 h. Only adult animals (>100 g) were used in the experiments.

In the laboratory, tuco-tucos from experiment 2 were directly transferred to the experimental condition, and those in experiments 1 and 3 were previously maintained for at least 7 days in a room with minimum noise and natural photoperiod provided by a glass window. Relative humidity ranged from 30 to 60% and room temperature was maintained at 24 ± 2°C, which is within the thermoneutral zone for this species (Tachinardi et al., 2017). Data loggers (HOBO U10/003, Onset Computer Corporation, Bourne, MA) recorded room temperature and relative humidity every 15 min. Animals were individually housed in a cage with wood shavings for bedding and equipped with an activity wheel (23 cm in diameter, 10 cm wide, 1 cm between the bars) connected to a data acquisition system which recorded wheel revolutions at 5-min intervals (ArChron Data Acquisition System—Simonetta System, Universidad Nacional de Quilmes, Buenos Aires, Argentina). Food (carrots, sweet potatoes, native plants, sunflower seed and commercial rabbit pellets) was provided *ad libitum*, with daily replacement at random times.

During laboratory experiments, animal cages were maintained inside opaque insulation boxes, equipped with dedicated systems for ventilation and lighting. Incandescent red-light bulbs provided continuous dim red light (1–5 lux) to facilitate animal care. A fluorescent bulb (200–250 lux at cage level) was turned on and off by a timing device to control the light (L) and dark (D) regimens. Each isolation box held up to 4 acrylic cages (53 × 29 × 27 cm). Animals used in experiment 3 were also surgically implanted with temperature sensitive transponders (G2 E-Mitters, Mini-Mitter, Bend, OR) to allow for semi-continuous monitoring of core temperature (T_*b*_; for surgical details, see Tachinardi et al., 2014). Data from the intra-abdominal transmitter were collected at 5-min intervals by a receiver (ER 4000, Mini-Mitter, Bend, OR) placed below the cage and processed using the software VitalView (Mini-Mitter, Bend, OR).

### Field Recordings (Experiment 1)

We used three semi-natural outdoor enclosures built in an area with native vegetation (enclosure 1: 10 × 5 × 1 m; enclosures 2 and 3: 12 × 6× 1.5 m). Enclosures were surrounded aboveground by wire mesh fencing and 1 m deep underground by concrete blocks to prevent tuco-tucos from escaping (Supplementary Figure 1). A nylon mesh also covered each enclosure to prevent aerial predation. Since native vegetation was enough for foraging, no extra food was provided.

Animals were released in the semi-natural enclosures with biologgers that recorded their activity and light exposure. From January to March (summer season) of 2015, 2016, and 2017, 19 freshly caught tuco-tucos (145.5 ± 37 g; 8 males; 11 females) were released individually inside each enclosure for different deployment durations (*n* = 15 short term recordings of 6–24 days; *n* = 4 long term recordings of 100–152 days). Deployment duration in the field enclosures was adjusted along the experiment, considering the trade-off between long duration recordings and increasing chance of death, predation or escape of animals with time. Both light and activity biologgers were mounted on a collar, built with cable ties inserted through silicon tubing, and deployed on tuco-tucos. Light loggers (15 × 6 × 6 mm; weight 0.65 g; model W65, Migrate Technology, United Kingdom) detected bouts of surface emergence during daylight within civil twilight limits, with no sensitivity to moonlight. Light intensity in the range of 1–19,000 lux (resolution of 249 discrete levels) was recorded every 5 min. In addition, accelerometers (23 × 12 × 10 mm; weight 2 g; model Axy-3, Techno Smart, Italy) were used in 6 individuals to record daily rhythm of general activity. Acceleration in three spatial axes (XYZ) was recorded in the range of −4 to +4 G-forces (8 bits resolution) every second. Activity was extracted from raw data by calculating the Vectorial Dynamic Body Acceleration (VeDBA) as in Qasem et al. (2012).

We named **field-activity duration** the interval between activity onset and offset measured in the field. Field-activity duration was measured in the accelerometry data from 3 animals recaptured in summer in the current work and another 3 animals in autumn/winter from Jannetti et al. (2019). The fourth summer animal with activity data did not show stable activity rhythms during the field condition, thus its data could not be used in this analysis. For each of the 6 accelerometry recordings, a subset of 10 days was extracted, based on the robustness of activity/rest rhythm. Onset and offset of activity phase for each day were determined through El Temps software eye-fitting tools (Díez-Noguera, 2020). Field-activity duration was calculated for each day and it was averaged by animal. Comparison between averaged values from summer and winter season was performed with one-sided Student’s *T*-test (de Winter, 2013). This analysis as well as the next ones described were performed in R software (R Core Team, 2020), unless specified otherwise. The first 24 h of both sensors’ data were excluded from all analyses.

Soil temperature was recorded by a buried temperature logger (HOBO^®^, Onset Computer Corporation, Bourne, MA; accuracy ± 0.5°C from 0 to 50°C) at 20 cm deep (**T_*soil*_**). Data from external temperature at the surface (Jannetti et al., 2019) were obtained using a temperature logger inserted inside a taxidermied tuco-tuco exposed to the sun, to account for radiation and convection effects. This measurement is called operative temperature (T_*e*_) (Chappell and Bartholomew, 1981). Due to technical difficulties, T_*e*_ data could not be collected for all field recordings, thus, we used an estimation of T_*e*_ from a semi-continuous satellite dataset to cover the entire experiment duration. This was possible due to numerous factors that diminished the microenvironmental variations present in our T_*e*_ measures: the region studied has sparse vegetation and low retention of humidity; T_*e*_ data were summarized in 3-h averages; and measures were taken in open areas, where tuco-tucos usually emerge to the surface. Average earth surface “skin” temperature (T_*skin*_) measured by satellite (Jin and Dickinson, 2010) was obtained from GLDAS_NOAH025_3H v2.1 dataset (Beaudoing and Rodell, 2020). The dataset is available in the Giovanni online data system, developed and maintained by the NASA GES DISC (Acker and Leptoukh, 2007). T_*skin*_ detects radiation emission from the earth surface, discounting the effect of atmosphere. This dataset collected data averaged every 3 h for each point of a grid with 0.25 degrees resolution (approximately 760 km^2^). T_*e*_ was estimated through a linear regression model as a function of T_*skin*_ and season (categorical variable with levels “summer,” “autumn,” “winter,” and “spring”). The resulting R^2^ of 87% of the regression model was considered sufficient to replace T_*e*_ by the predicted values of the model (Supplementary Figure 2). The new temperature variable without gaps was named surface temperature (**T_*sur*_**).

### Statistical Analysis of Field Data (Experiment 1)

Statistical models can disentangle the relative contributions of candidate environmental factors that modulate biological variables in the field (Kowal et al., 1976; Bennett, 1987). Generalized linear mixed models were used to test the role of environmental temperatures in modulating tuco-tucos surface emergences during the photophase, in summer and winter separately (glmmTMB function of “glmmTMB” package; Brooks et al., 2017). Counts of surface episodes every 3 h were considered as the response variable. Surface episodes were defined as events in which light-loggers detected Illuminances higher than 2 lux, based on the minimum sensitivity of the loggers and on previous observations that light loggers do not detect light when tuco-tucos are inside tunnels. The following independent variables were included in both models: T_*soil*_ (soil temperature at 20 cm below ground); T_*sur*_ (surface temperature described above); Hour [5 categories representing 3 h blocks: A (06:00–08:55 AM); B (09:00–11:55 AM); C (12:00–02:55 PM); D (03:00–05:55 PM); E (06:00–08:55 PM)]. For winter data, Hour categories A and E were excluded, since more than 50% of these blocks corresponded to nighttime, when light loggers do not detect emergences. The final model was chosen from a biologically plausible initial set, based on lowest Akaike Information Criterion (AIC) values (Zuur et al., 2009). The variables included had variance inflation factors (VIF) (Zuur et al., 2009) lower than 5. A total of 20 extreme values of T_*soil*_ and T_*sur*_ (more than 2 standard deviations or less than −2) were underrepresented in the datasets and were excluded to avoid bias, resulting in a sample size of 973 observations from 12 animals in the current summer data and 227 observations from 9 animals in the winter data published previously (Jannetti et al., 2019).

For summer data, surface emergence during the photophase had significant temporal dependence (Potvin et al., 1990), i.e., outside the 95% confidence interval of the autocorrelation function (acf function from “forecast” package) (Hyndman et al., 2020). Since autocorrelation violates the assumption of independence of the model, it was accounted for by adding an auto-regressive structure of order 1 (AR-1, Zuur et al., 2009), modeled as a function of Hour and allowing for this effect to be different according to the animals’ ID number (ID). A random intercept according to ID was not included in the summer model due to its negligible variance (6.841e-08), as in Pasch et al. (2013).

Although our datasets had a much finer time resolution, the choice to use five blocks (factors) in the “Hour” variable took into account that a higher number of factors would increase the number of regression parameters to be estimated and this would diminish the significance of the model, unless we increased the sample size (Zuur et al., 2009). At the same time, using fewer blocks, e.g., day vs. night only, would not give us enough time resolution to probe the varying effects of T_*sur*_ and T_*soil*_ along the day. Alternatively, we could have analyzed each time block separately with an independent model, eliminating the need for an “Hour” variable. However, that would have worsened the estimation of the other variables’ effects, since the sample size to each analysis would have been five times smaller.

### Exposure to Constant Darkness in Freshly Caught Animals (Experiment 2)

Freshly caught tuco-tucos were brought to the laboratory in summer and winter to evaluate aftereffects of natural entrainment. Captures were held in 2016 and 2017, between January 12th and February 1st for summer and between July 8th and 26th during winter, which implies that, in both seasons, the associated solstice had already occurred. After each capture, animals were taken to the laboratory (located at a walking distance), where they were weighed and sexed and then immediately released into constant conditions to enable measurement of aftereffects. The average mass of the 35 analyzed animals was 148 ± 55 g, including 16 females, 18 males, and one of undetermined sex. Running-wheel activity was measured under constant darkness (dim red lights—5 lux).

Data were plotted in actograms in the software El Temps (Díez-Noguera, 2020). In each actogram, activity onset and offset times were eye-fitted from the first 10 days in DD and lab-activity duration (α) was calculated as the difference between them. Significant differences were evaluated using Student’s *T*-Test.

### Exposure to Constant Light (Experiment 3)

To evaluate splitting in activity and temperature rhythms, nine tuco-tucos (6 females and 3 males) were subjected to the following conditions: first, LD 12:12 (lights on at 07:00) for 13 days; then, LL (200–300 lux) for either (i) 92 days (individuals #69, #99, #100, # 101, # 102, # 104, and # 106) or (ii) 145 days (individuals # 46 and # 52). The sample size was chosen based on previous reports of splitting occurrence in at least half of both male and female tuco-tucos exposed to prolonged LL (Valentinuzzi et al., 2009). Wheel-running and body temperature of all individuals were monitored at 5-min intervals. Data were plotted in actograms for visual analysis and chi-square periodograms (Sokolove and Bushell, 1978) were performed to evaluate periodicities in the data. Splitting events were recognized by visual inspection of the actograms when there was more than one activity phase per day and by the periodogram when periods close to 12 h were detected. Analyses were performed using the software El Temps (Díez-Noguera, 2020).

### Mathematical Modeling

The two-oscillator model of the circadian clock proposed by Pittendrigh and Daan (1976c) suggests that the clock is composed of a morning (M) oscillator and an evening (E) oscillator. We simulated this model for the circadian clock of tuco-tucos, with Pavlidis-Pittendrigh oscillators. The equations were used in our previous studies of the entrainment of M and E oscillators to regular photoperiods (Flôres and Oda, 2020) and the splitting phenomenon in hamsters in constant light (Oda and Friesen, 2002). State variables and parameters of M and E are indicated in the equations below. The terms for each oscillator are identified by subscribed letters.

Morning oscillator (M):


dRMdt=RM-cM⁢SM-bM⁢SM2+(dM-L)+KM

$$\frac{{\mathrm{dS}}_{M}}{\mathrm{dt}}=R_{M}-a_{M}\,S_{M}+C_{\mathrm{EM}}\,S_{E}$$

Evening oscillator (E):


dREdt=RE-cE⁢SE-bE⁢SE2+(dE-L)+KE

$$\frac{{\mathrm{dS}}_{E}}{\mathrm{dt}}=R_{E}-a_{E}\,S_{E}+C_{\mathrm{ME}}\,S_{M}$$

Briefly, *R* and *S* state variables describe the phase of the oscillator at each time point. *R* is prevented from assuming negative values (*R* > 0). *a*, *b*, *c* and *d* are fixed parameters that compose an oscillator configuration, with intrinsic period, amplitude and phase-response. We assigned the following configuration to the oscillators: *a* = 0.85, *b* = 0.3, *c* = 0.8, *d* = 0.5. *L* is the light variable set to 0 to represent darkness and changed to 1.1 arbitrary units for 1 h to make a light pulse. The non-linear term *K* smooths the numerical integration [*K* = *k*_1_ / (1 + *k*_2_*R*^2^), *k*_1_ = 1, *k*_2_ = 100]. Finally, *C*_*EM*_ indicates the coupling strength of the E oscillator onto the M oscillator and *C*_*ME*_ defines the opposite coupling strength. The coupling parameters were assigned a symmetrical value: *C_EM_* = *C_ME_* = 0.03. These model parameters were chosen based on our previous work with the equations (Flôres and Oda, 2020). They generate a model with a free-running period close to 24 h and that is responsive to skeleton photoperiods. Computer simulations were performed in the CircadianDynamix extension of the Neurodynamix II software (Friesen and Friesen, 1994), using numerical integration at 1,000 steps per 24 h, with the Euler method.

The M-E model was exposed to daily light-pulse regimens that mimic the light exposure patterns of tuco-tucos in the field. In each simulation, the oscillator system was left in constant darkness for 20 days, followed by the light pulse regimens for 50 days, and constant darkness again for another 20+ days. To mimic the irregular light exposure of tuco-tucos, daily light pulses were applied at random times, uniformly distributed in pre-defined time-intervals within the photophase. Random pulse times were generated in the software R (R Core Team, 2020), as described in the Supplementary Text. Three light pulse regimens of increasing complexity were defined, to probe different features of the tuco-tucos light exposure, as described below.

Model I—one single pulse per day that can occur at any random time throughout the photophase. It is the simplest light exposure model, with minimal information on the timing of the photophase.

Model II—two pulses per day, one constrained to the first half of the photophase and the other pulse to the second half. The extra pulse adds more timing information about the photophase.

Model III—two pulses per day each constrained to a 4 h-interval phase-locked to one of the simulated twilights. This is still simplified, but it adds yet more timing information on the photophase, by replicating a feature observed in tuco-tucos: twilight exposure in summer, and noon exposure in winter.

In each of the models, the photophase onset and offset times were then systematically varied to simulate short and long-day photoperiods, with photophase durations varying from 8 h (LD 8:16) to 16 h (LD 16:8). Each light pulse was constrained to input only the M or E oscillator, depending on whether the pulse occurred in the first or in the second half of the photophase, respectively. This strategy successfully replicated experimental results of nocturnal rodents exposed to “skeleton” photoperiods (Flôres and Oda, 2020).

The phase relationship between E and M (ΔΦ_*EM*_) was calculated on each day during the light pulse regimens and also in constant darkness after the pulses, to verify aftereffects of entrainment to the different photoperiods. We first determined the reference phases for each oscillator, as the maximum of the state variable *S* within each cycle. To obtain the ΔΦ_*EM*_ on a given day, we measured the absolute difference between the time of the E reference phase and the time of the M reference phase. Mean and standard deviation of ΔΦ_*EM*_ were calculated in the last 20 days under each light pulse regimen.

## Results

### Experiment 1: Natural Daily Light Exposure and Activity in Summer and Winter

From the 19 animals deployed in the field enclosures in summer, 13 were successfully recaptured (153 ± 39 g; 7 males; 6 females). Four of the recaptured tuco-tucos (169.7 ± 46 g; 2 males; 2 females) had collars containing accelerometers. Data from 9 animals in winter and 1 animal in autumn were taken from Jannetti et al. (2019) and used for comparisons between seasons.

**FIGURE 1:**
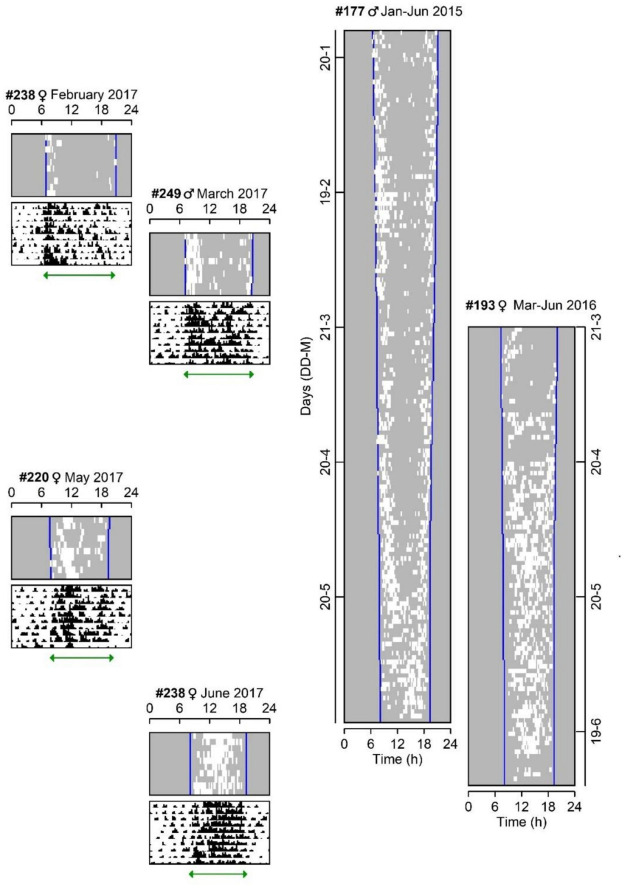
Daily light exposure and activity patterns of individual tuco-tucos released in semi-natural enclosures from summer to winter. Actograms are positioned according to the annual timescale. The duration of the recordings was different for each animal. Left: Four shorter recordings (10 days), from individuals released in the enclosures throughout 2017, showing light exposure (white marks) and simultaneously recorded general activity (black marks). Green horizontal arrows indicate field-activity duration of each animal—#238 (Feb): 13.8 ± 1.7 h; #249 (Mar): 14.1 ± 0.8 h; #220 (May): 12.3 ± 0.5 h; #238 (Jun): 11 ± 0.5 h. Summer and winter field-activity durations were statistically different (*n* = 6, Student’s *t*-test *p* = 0.02). Right: Two individuals with long term recordings of light exposure (white marks)—#177 from January to June 2015 and #193 from March to June 2016. Light exposure records indicate the time of surface emergences during sunlight hours (between civil twilight limits—blue vertical lines). Data of animals #177, #193, #220, #238 were taken from Jannetti et al. (2019).

**FIGURE 2:**
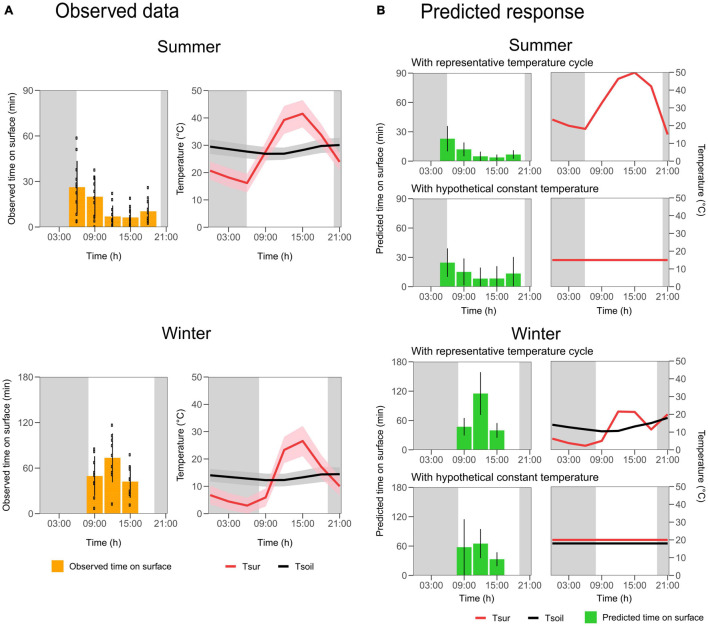
Observed and predicted time tuco-tucos spent on the surface during the light hours of the day, in summer and winter. **(A)** Left: Observed time spent on surface according to collar light-logger data. Means by animal (black points) for every 3 h were averaged to obtain the overall pattern (yellow bars). Right: Mean and standard deviation (lines and shaded areas) of T_*sur*_ (red) and T_*soil*_ (black) every 3 h, during animals’ recordings. **(B)** Predicted overall pattern of time on surface (green bars) according to the final generalized linear models fitted for summer (above) and winter data (below) assuming two different scenarios: representative natural temperature variation and hypothetical constant temperatures. Vertical lines in the bars: standard deviation of the predictions for each individual. T_*sur*_ (red lines) and T_*soil*_ (black lines) used for predictions are shown to the right of the corresponding graph. T_*soil*_ is not shown for summer since this variable was not significant in the model.

Light-logger data indicated when the subterranean tuco-tucos were on surface during daylight and, consequently, the timing of their exposure to light. Daily surface emergences and light exposure occurred near the twilights during mid and late summer (Figure 1 and Supplementary Figure 3), contrasting with the pattern in winter (Flôres et al., 2016; Jannetti et al., 2019), when both were concentrated in the middle of the day (Figures 1, 2A). The two animals with longer deployment duration (Figure 1 #177 and #193) show the gradual transition between these two contrasting patterns during autumn.

Accelerometer data, on the other hand, informed the timing of general activity, either on surface or underground, based on its sensitivity to body movement. As seen in Figure 1, general activity is organized in activity bouts that occur both during the day and night, with highest levels during the day. This occurs for both summer and winter data. Furthermore, when accelerometer data are compared to light-logger data, surface emergence roughly coincides with 47% of the highest levels of general activity in autumn/winter (Jannetti et al., 2019). Using the same calculations, surface emergences occur in 20% of highest activity of tuco-tucos in summer. Daily field activity duration was significantly greater for summer animals (*n* = 3, current dataset) than for late autumn or early winter animals (*n* = 3, from Jannetti et al., 2019) (2.13 h difference between means; Student’s *T*-test *t* = −4.8, *p* = 0.02, *df* = 2.2).

We next used statistical models to verify the role of soil (T_*soil*_) and surface (T_*sur*_) temperatures in stimulating or inhibiting the surface emergence during the day of tuco-tucos in summer and winter. We fitted two Negative Binomial generalized linear mixed models, for summer and winter data separately. They were selected from putative models with different combinations of environmental measures (Table 1). In the final summer model, T_*sur*_ had a significant contribution to explain surface emergences, showing a negative correlation to the counts of emergence episodes during the photophase (estimated coefficient of −0.7 ± 0.1, *p* < 0.001), while T_*soil*_ had no significant effect. In the winter model, however, both T_*sur*_ and T_*soil*_ had significant contributions. T_*sur*_ showed a positive correlation with emergence episodes (0.5 ± 0.2, *p* = 0.003), and T_*soil*_, a negative correlation according to Hour (time of day) (no significant effect at 09:00–11:55 AM; −0.5 ± 0.2 at 12:00–02:55 PM, *p* = 0.01; −0.4 ± 0.2 at 03:00–05:55 PM, *p* = 0.03).

**TABLE 1:** Comparison between generalized linear models built to explain the counts of surface episodes every 3 h in summer and winter, using different combinations of the environmental measures.

Summer			
Fixed effect variables	**AIC**	***X* ^2^**	***R*^2^ (%)**
Hour+T_*sur*_	**3901.2**	**61.2**	**55.9**
Hour+T_*sur*_+T_*soil*_	3906.4	61.3	55.9
T_*sur*_+Hour*T_*soil*_	3906.6	76.1	56.9
Hour*T_*sur*_+T_*soil*_	3909.3	67.9	56.2
Hour*T_*sur*_+Hour*T_*soil*_	3911.6	79.1	56.9
Hour+T_*soil*_	3929.5	34.0	53.6
Hour	3935.5	22.4	52.4

**Winter**			

Fixed effect variables	**AIC**	***X* ^2^**	***R*^2^ (%)**
Hour*T_*soil*_+T_*sur*_	**1517.7**	**42.2**	**45.9**
Hour+T_*soil*_+T_*sur*_	1518.2	34.8	42.6
Hour+T_*sur*_	1518.7	30.0	42.7
Hour*T_*sur*_+Hour*T_*soil*_	1519.0	45.2	47.6
Hour	1522.4	22.5	41.9
Hour*T_*soil*_	1522.6	33.1	49.9
Hour*T_*sur*_+T_*soil*_	1523.0	34.9	42.4
Hour+T_*soil*_	1523.6	25.4	46.1
T_*soil*_	1532.1	7.7	45.4
T_*sur*_+T_*soil*_	1534.2	11.6	44.8
T_*sur*_	1539.5	2.2	34.7

*Asterisks represent interaction effect between variables. Models with interactions included the corresponding isolated terms. The final model (bold values) was chosen by lowest AIC (Akaike Information Criterion, see section “* *Materials and Methods* *”* *). X^2^* *-* *values were calculated against the null model. R^2^* *-* *values account for the random structure. Datasets had 973 observations and 12 animals for summer and 227 observations and 9 animals for winter.*

These findings in the winter data agree with the analysis performed in Jannetti et al. (2019) in the sense that T_*soil*_ had a significant negative correlation with emergence episodes, although interaction between T_*soil*_ and Hour was not considered before. The inclusion of this interaction here did not improve the model greatly: compared to the winter model without T_*soil*_-Hour interaction, the model with the interaction decreased less than 2 AIC units (Table 1; Bolker, 2007) and the difference between the models was only significant at the 5% level of significance (*X*^2^-test, *p* = 0.02). However, the T_*soil*_-Hour interaction revealed that surface emergences do not correlate with T_*soil*_ in the morning, while the correlation is significantly negative in the afternoon. These results indicate that T_*soil*_ does not modulate the onsets of tuco-tucos surface emergences in the morning. On the other hand, they do not exclude the possibility of modulation of the offset of emergences in winter.

Contrary to the current winter results, our previous analysis of the autumn/winter data (Jannetti et al., 2019) had not shown an effect of surface temperature on the tuco-tucos surface emergences. Part of this divergence may be due to different techniques used to measure surface temperature (temperature logger vs. satellite measurements). Additionally, the divergent results may be due to the inclusion, in the previous work, of autumn and winter animals in the same model. Notably, our current analysis reveals a seasonal variation in the role of T_*sur*_, with opposite effects in summer and winter. In this sense, pooling together autumn and winter data in the previous analysis might have caused an underestimation of the effects of surface temperature on tuco-tucos’ surface emergence (Jannetti et al., 2019).

Integrating all variables’ effects, surface emergence episodes in summer are less dependent on exogenous temperature conditions than in winter, since the summer crepuscular pattern can be deduced without the T_*sur*_ effect, as can be seen in the scenario of constant temperature in Figure 2B. Despite a significant T_*sur*_ effect detected in our analysis, there is a rhythmic component in the summer surface emergences (details in the methods), not fully explained by T_*soil*_ or T_*sur*_ effects. These results suggest that endogenous factors may have a more important role to generate this emergence pattern rather than other environmental variables not considered here.

Our data support the seasonal role of exogenous temperature in modulating surface emergences in tuco-tucos. However, the surface activity is only one behavioral component of the daily activity-rest rhythm, which also includes what the animals do underground throughout day and night. We observed a significant difference in the daily field-activity duration of the general activity, between summer and winter from the accelerometer data (Figure 1). In other words, not only surface activity but the gross activity (surface+underground) was shown to have different duration between the seasons. This seasonal change could be generated by masking effects of environmental cycles, but it could also be due to changes in the endogenous regulation by the circadian clock. Moreover, the analysis of surface emergences alone suggests an endogenous contribution to the patterns observed, mainly in summer. Thus, in the next experiment, we tested the role of endogenous circadian timing in the regulation of seasonal activity.

### Experiment 2: Aftereffects of Natural Photoperiods

New sets of freshly caught animals were brought to the laboratory, to evaluate the endogenous contribution to the observed seasonal variations in activity patterns. We used a standard experiment in chronobiology which consists of releasing synchronized organisms into constant laboratory conditions, to eliminate exogenously generated rhythmicities. In the first days under this condition, before their rhythms start to free-run, they display “aftereffects” of previous entrainment (Pittendrigh and Daan, 1976a; Tackenberg et al., 2020). The aftereffects indicate rhythmic parameters that have been encoded in the endogenous circadian clock.

**FIGURE 3:**
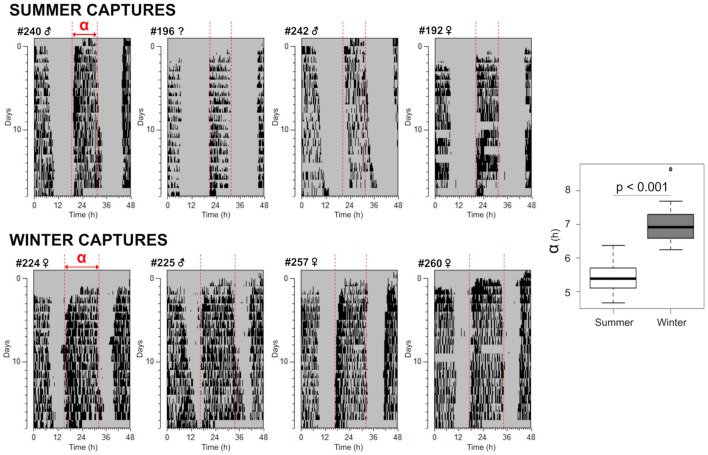
Aftereffects of natural photoperiods in tuco-tucos captured in summer and winter and transferred to lab DD. Representative double-plotted actograms of summer- **(upper panel)** and winter-caught **(lower panel)** tuco-tucos under DD. At the right, boxplots illustrate the duration of daily lab-activity (α) displayed during the aftereffects of natural entrainment, compared between summer (*n* = 17) and winter (*n* = 18) animals.

Animals captured during summer and winter were immediately released into DD conditions to evaluate aftereffects in the lab-activity duration (α). Representative actograms are presented in Figure 3, with the mean α of 5.4 h (*n* = 17) for summer and 7.0 h (*n* = 18) for winter animals (Student’s *T*-test *t* = −8.8907, *df* = 32.086, *p*-value = 3.621e-10). As an important remark, tuco-tucos are nocturnal in the lab and diurnal in the field (Tachinardi et al., 2017). For this reason, field-activity duration is inversely proportional to lab-activity duration α, as seen comparing Figures 1, 3, in summer and winter.

The results confirmed seasonal α differences when animals were free from any masking effect, in laboratory DD conditions. These α aftereffects indicate a seasonal change in the endogenous control of activity, reminiscent of season-dependent entrainment of the circadian clock by different photoperiods. Persistent aftereffects in α in DD are explained in terms of a slowly relaxing coupling between the component oscillators within the SCN. Thus, the following experiment tests the multi-oscillatory composition of the circadian clock of tuco-tucos.

### Experiment 3: Splitting of Rhythms Under Constant Light

**FIGURE 4:**
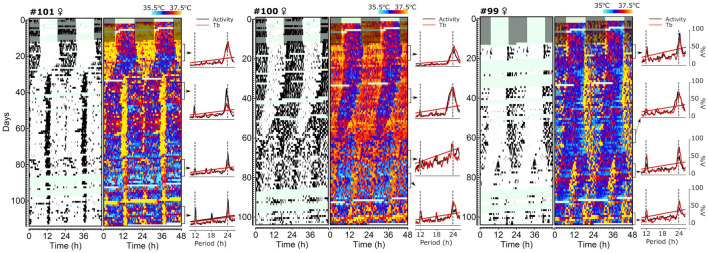
Splitting of wheel running and body temperature rhythms in tuco-tucos under constant light conditions. Three panels depicting data from representative individuals: Animal #101 **(left)**, Animal #100 **(middle)**, and Animal #99 **(right)**. In each panel, the left double-plotted actogram depicts wheel running (black bars indicate when running was recorded) and the actogram to the right depicts body temperature (a color gradient is used to display temperature values, maximum and minimum values are indicated in the legend above the graph). Areas shaded in gray indicate the dark phase of the LD cycle which preceded constant light. Areas shaded in light green indicate missing data. Graphs to the right of actograms depict results of the chi-square periodograms (Sokolove and Bushell, 1978) for wheel running (black) and temperature (red) calculated for the data corresponding to the intervals indicated by the arrows and brackets. Values are shown as spectral power (percentage of variance) as a function of the period tested (5-min steps were used). Inclined lines in the periodograms indicate the significance thresholds (*p* < 0.05). 24-h and 12-h periods are emphasized by the dashed vertical gray lines for reference.

We next evaluated the splitting phenomenon (Pittendrigh and Daan, 1976c) in tuco-tucos under LL to probe the two-oscillator composition of their circadian clock. Splitting occurred in seven out of the nine animals (77, 7%), displaying great variability of splitting patterns (Figure 4 and Supplementary Figure 4). The splitting happened gradually in three individuals (#46, #100, and #104) and abruptly in four (#52, #99, #101, #102). Preceding splitting, the free-running period of the rhythms (τ) shortened in two animals (#52, #100), lengthened in two (#102 and # 104) and remained stable in three (#46, #99, and #101).

Rhythmic patterns of wheel-running and body temperature were similar in all but one of the animals that experienced splitting (Supplementary Figure 4). The exception was individual # 52 in which wheel running became arrhythmic 10 days after the splitting occurred. During the days when wheel running was arrhythmic, a third component, with a τ of 25.2 h, could be detected in the body temperature rhythm and became undetectable after wheel running rhythmicity was reestablished.

The finding of multiple components in activity and temperature rhythms under LL is an evidence that the underlying circadian pacemaker of tuco-tucos is composed of multiple oscillators. Next, we use mathematical modeling to test whether a minimal two-oscillator model could account for the seasonal adjustments in activity α, when exposed to light regimens that mimicked the light exposure of tuco-tucos in summer and winter.

### Mathematical Modeling

**FIGURE 5:**
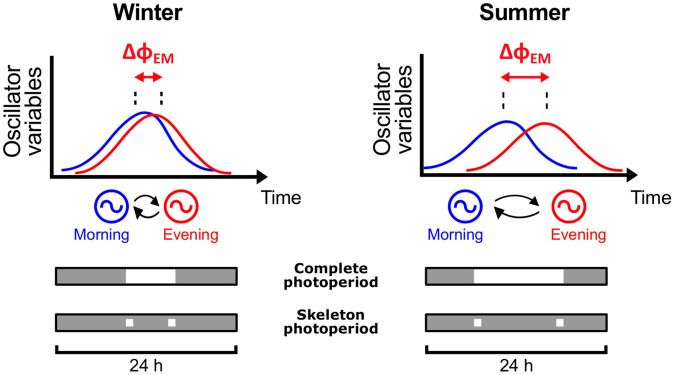
Schematic representation of the two-oscillator model of the circadian clock and its role in photoperiod encoding. The model proposed by Pittendrigh and Daan (1976c) consists of a morning (M) oscillator that tracks dawn and an evening (E) oscillator that tracks dusk. M and E (circles) are coupled to each other (curved arrows). In the upper graphs, blue and red curves represent the state variables of M and E, respectively. As the photoperiod changes from short days in winter to long days in summer, there is a change in the phase relationship between E and M (ΔΦ_*EM*_, red horizontal arrows), which modifies the state of the circadian clock. Below the graphs, gray/white bars represent the light/dark times of the LD cycles. Upper bars indicate an LD cycle with complete photoperiod, i.e., light occurring during the complete photophase. Lower bars represent skeleton photoperiods, a simplified experimental protocol that reproduces the effects of photoperiod with only two light pulses, applied at the twilights.

In mammals, the morning (M) and evening (E) oscillators (Pittendrigh and Daan, 1976c) are thought to be composing parts of the SCN. It is predicted that, as the photoperiod changes, the M oscillator tracks dawn and the E oscillator tracks dusk, resulting in adjustments of the phase relationship between them (ΔΦ_*EM*_) (Figure 5). In turn, adjustments in ΔΦ_*EM*_ are reflected in activity α, since the SCN control the circadian rest-activity rhythm.

Our hypothesis is that the tuco-tucos seasonal light exposure acts on this photoperiod-encoding mechanism, generating the observed endogenous adjustments in the tuco-tucos daily activity α at different seasons. The hypothesis is supported by our current findings that (i) light exposure patterns in tuco-tucos are different under different photoperiods; (ii) seasonal aftereffects in α are seen in animals brought from the field directly into laboratory constant conditions, which suggests seasonal adjustments of the circadian clock; (iii) splitting of tuco-tucos activity/rest rhythms into two components, under constant light in the laboratory, indicates a multi-oscillatory circadian clock.

**FIGURE 6:**
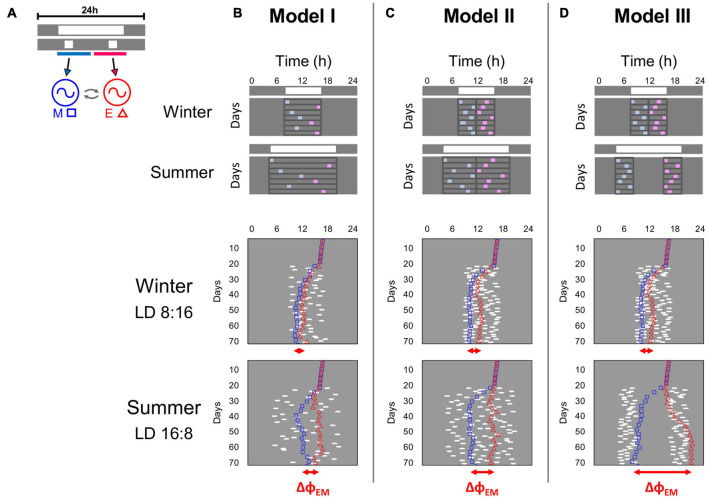
A mathematical model was used to simulate 3 scenarios of light exposure at different seasons, and their impact on the synchronization of a two-oscillator model of the circadian clock. **(A)** Morning (M) and Evening (E) oscillators (circles), mutually coupled (curved arrows), were exposed to daily light pulses at random times within the photophase. The 24 h light regimen is represented by gray (dark) and white (light) bars. Upper bar: complete photoperiod. Lower bar: light exposure episodes (light pulses). Pulses between light onset and midday were applied to M (blue line and arrow), and pulses from midday to light offset were applied to E (pink line and arrow). **(B–D)**—Light exposure models I, II, and III, in winter and summer photoperiods. Upper panels: Schemes of the light pulse schedules. Gray/white bars depict the complete photoperiods and conceptual schemes represent the light exposure episodes along consecutive days, with blue and pink squares indicating light pulses applied to M and E, respectively. Lines around the light pulses on each day delimit the distribution interval of the pulses. Lower panels: Actograms illustrate the model dynamics on consecutive days under the light pulses (white marks). Colored symbols depict the reference phases of M (blue squares) and E (red triangles). Oscillator symbols are plotted only every third day for better visualization. Below the actograms, red horizontal arrows show the average ΔΦ_*EM*_ on the last 20 days. For details on the light exposure models, see main text. For model parameters see section “Materials and Methods.”

To test the hypothesis, two limit-cycle oscillators representing the M-E model were computer simulated (Figure 6A). The oscillators were exposed to simplified light cycles that mimicked the observed natural light exposure of tuco-tucos in winter and summer, with increasing complexity from Model I to Model III (Figures 6B–D, upper panels) (details in section “Materials and Methods”). Changes in ΔΦ_*EM*_ were used as a proxy for α and as an indication of the efficacy of these light signals in informing day length.

**FIGURE 7:**
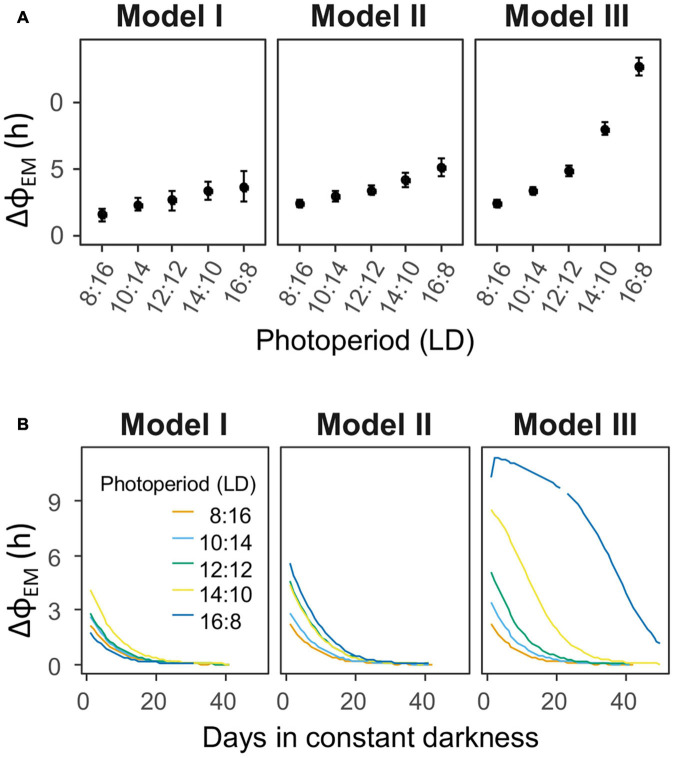
Quantifications of the phase relationships between E and M (ΔΦ_*EM*_) in the 3 models, during the light exposure, and afterward in constant conditions. **(A)** Average ΔΦ_*EM*_ in the last 20 days of light pulses, under different photoperiods. **(B)** Progression of ΔΦ_*EM*_ along the days in constant conditions (DD), after termination of the pulses. The initial values in constant conditions reveal aftereffects of the previous entrainment to the distinct photoperiods.

The dynamics of M and E under the different light exposure models are reported for 2 extreme photoperiods in Figures 6B–D (lower panels). Results for intermediate photoperiods can be found in Supplementary Figure 5. Despite the simplicity of Model I, with a single light pulse per day, it already presented a modest adjustment in ΔΦ_*EM*_, in short days vs. long days (Figure 6B, lower panels). The average ΔΦ_*EM*_ was 1.6 h in the short “winter” days (LD 8:16), and 2.7 h in the long “summer” days (LD 16:8), with intermediate values for the other photoperiods (Figure 7A). Upon termination of the pulses, in constant conditions (DD), ΔΦ_*EM*_ gradually decreased to the steady state value (Figure 7B). The initial values in DD were reminiscent of the previous entrainment to the different photoperiods, which suggests aftereffect in ΔΦ_*EM.*_ The greater the initial value, the longer it took for the model to reach its steady state.

Tuco-tucos are exposed to more than one single light pulse per day in the field, thus, in Model II, we applied two pulses each day. The effect of photoperiod (Figure 6C, lower panels) was more pronounced than Model I, with ΔΦ_*EM*_ ranging from 2.4 h under short winter days to 5.2 h under long summer days (Figure 7A). When the pulses were turned off, in DD, there was once again a gradual adjustment of ΔΦ_*EM*_, tending toward the steady-state null value (Figure 7B). In contrast to Model I, however, there were larger initial differences in ΔΦ_*EM*_ between the photoperiods, which resulted in a longer duration of summer aftereffects (LD 16:8) in Model II.

In the field data, summer light exposure is concentrated in the twilights, while winter light exposure is concentrated around noon (Figure 1). This feature could contribute even further to the photoperiodic regulation of the circadian system. To test this hypothesis, we devised Model III, in which the pulses are concentrated in 4 h windows locked-on to the twilights. In Figure 6D (lower panels) we can see a greater difference between summer and winter synchronization in Model III, compared to the other two models. ΔΦ_*EM*_ ranged from 2.4 h in the summer to 12.7 h in the winter (Figure 7A). These large differences were also reflected in DD, resulting in a much longer duration of the aftereffects in ΔΦ_*EM*_ (Figure 7B).

## Discussion

Activity patterns of wild rodents have long been monitored through population-wise observations in natural habitats. In long-term field studies, changes in activity time, particularly the onset and offset of populational activity rhythms, are correlated with changes in photoperiod (Kenagy, 1976). When individual wheel-running rhythms of some rodent species were measured under systematic variation of artificial photoperiods, the changes in activity onset and offset often reproduced those observed in field populations (DeCoursey, 1972; Elliott, 1976). Still in the laboratory, more realistic settings with simulated burrows have also been developed to allow partial expression of natural light exposure patterns (DeCoursey, 1986; Pratt and Goldman, 1986). By means of biologging devices, individual rhythms can also be measured directly in the field, where animals express natural behaviors that are precluded in lab cage studies (Williams et al., 2014, 2016; Jannetti et al., 2019; Zhang et al., 2019, 2020; Silvério and Tachinardi, 2020). By measuring the activity of subterranean rodents in a semi-natural habitat with accelerometers and light-loggers, we were able to decompose the daily activity into two components, the general and the surface activity, a separation that would not apply to unsheltered, epigeous organisms. In nature, the surface component of activity also defines the active light exposure allowing us to get a picture of the light/dark information experienced by tuco-tucos and evaluate its consequences to circadian (Flôres et al., 2013, 2016) and seasonal rhythmicity. In this framework, we have addressed the question of whether tuco-tucos can get cues of photoperiod out of their irregular and self-imposed, daily pattern of light exposure. In the context of a subterranean rodent, this is indissociable from the question of which factors, exogenous and endogenous, drive their emergence from the underground to the surface.

### Temperature Is an Exogenous Factor Modulating Seasonal Changes in Tuco-Tucos’ Time on Surface

The acute effect of temperature on daily activity patterns has long been demonstrated for several diurnal rodent populations. Many rodent populations in nature present a bimodal activity distribution in summer, in contrast to a unimodal pattern in winter (Halle and Stenseth, 2000) and this seasonal change is attributed to avoidance of high midday heat loads in summer. Accordingly, summer activity is bimodal in Piute ground squirrels (*Spermophilus mollis*) and degus (*Octogon degus*) living in open habitats, as they retreat to shade spots or burrow entrances during midday. In contrast, those living in shrubby habitats, with access to the extensive shadows, still display activity in the summer midday (Sharpe and Van Horne, 1999; Kenagy et al., 2002; Bacigalupe et al., 2003). Similarly, summer activity is not bimodal in squirrel species with adaptive morpho-physiological and behavioral strategies that reduce heat load and increase heat dissipation (Chappell and Bartholomew, 1981; Fick et al., 2009). Together, these studies support the causal relationship between summer bimodal activity and the thermoregulatory pressure of high midday heat loads (Kenagy et al., 2002).

In contrast to the epigeous species that use burrows for temporary retreats from heat load (Hainsworth, 1995), subterranean tuco-tucos spend most of their time in the tunnels and just emerge to the surface in brief episodes, never wondering around (Tomotani et al., 2012). Thus, in principle, soil temperature could be more determinant than surface temperature in modifying activity patterns of subterranean rodents. Indeed, soil temperature was observed to modulate activity patterns in other subterranean rodent species (Rado et al., 1993; Vlasatá et al., 2017). Nevertheless, hot external temperatures have also been shown to curtail surface activity of subterranean and fossorial species in summer, generating a bimodal pattern of aboveground incidence (Rezende et al., 2003; Hinze and Pillay, 2006). We then hypothesized that both soil and surface temperatures could have a role in shaping emergence patterns of tuco-tucos during summer in a desert habitat.

We had previously shown differences in the time course of surface and soil temperature cycles in our study area, in northern Monte desert, due to the delay of daily heat flow in the soil (Jannetti et al., 2019). As a consequence, the highest soil temperatures occur at night when surface temperatures are the lowest, while, surprisingly, the lowest soil temperatures occur at noon when surface temperatures are the highest. In the previous autumn/winter records, surface emergence episodes occurred mostly at midday when not only it was warmest aboveground but also coolest below ground (Jannetti et al., 2019). The current results confirmed that, in winter, emergence episodes statistically correlate to lower T_*soil*_ and higher T_*sur*_. In summer, however, no effect of T_*soil*_ was observed on emergence episodes. On the other hand, T_*sur*_ had a significant inhibitory role, although the crepuscular pattern of time on surface could be deduced without the T_*sur*_ effect. Thus, to some extent, the model confirmed the masking role of surface temperature, which partly explains avoidance of midday hours and concentration in twilight times, as pointed out for other species (Kenagy et al., 2002; Bacigalupe et al., 2003; Rezende et al., 2003; Hinze and Pillay, 2006; Vlasatá et al., 2017). Nevertheless, the magnitude of the temperature effect was potentially lower than that encountered for some epigeous species (Long et al., 2005). This suggests that other factors, such as variable foraging demands and social interactions (Amaya et al., 2021), may also have an important role modulating timing of surface activity in tuco-tucos. Interestingly, while epigeous species retreat to shelters as a reaction to extreme surface temperatures (Chappell and Bartholomew, 1981; Long et al., 2005), subterranean tuco-tucos refrain from exiting to the surface if aboveground temperatures are too high in summer, being a subtly distinct behavioral response.

Taken together, our results point to a significant role of the daily ambient temperature cycle in masking surface activity of tuco-tucos. However, our model projects more subtle temperature effects in summer than in winter. The lack of association of summer emergences with T_*soil*_ and the fact that crepuscular patterns can be deduced disregarding T_*sur*_ effects suggest that other factors, e.g., the endogenous control, should contribute to driving the observed temporal pattern of emergences.

### Photoperiod Dependent Patterns of Suprachiasmatic Nuclei Entrainment Are the Endogenous Factors Driving Seasonal Changes in Time on Surface

The surface activity of a subterranean rodent is just one component of the general activity which also includes behaviors displayed underground, out of our sight. Accelerometers can detect body movements above or underground and they have revealed that, in both summer and winter, tuco-tucos display bouts of general activity throughout day and night (Figure 1). However, there is a predominant active phase during the day—an interval when activity bouts are concentrated—which allows definition of activity onsets and offsets. These allowed comparison of field-activity duration between summer and winter, which was significantly different. In other words, not only surface activity but the gross activity (surface+underground) was shown to have different onset and offset times between the seasons. The endogenous nature of this phenomenon was shown by releasing freshly captured tuco-tucos in winter and summer into constant laboratory conditions (Figure 3). They displayed clear aftereffects of natural entrainment, with summer and winter animals showing significantly different α. This is in accordance with previous reports in laboratory experiments with model organisms, using artificial LD cycles with different photoperiods (Pittendrigh and Daan, 1976a; Tackenberg et al., 2020). To our knowledge, this is the first time α aftereffects are demonstrated in organisms previously free-living in the field, as a result of natural entrainment in different seasons.

This result indicates that part of the seasonal changes in activity patterns are endogenous, likely due to different patterns of SCN entrainment by different photoperiods. The endogenous regulation means that we would likely observe seasonal changes in activity patterns even in the absence of the strong thermal constrains of the desert. Accordingly, seasonal variation of activity onset and offset times are also seen in species that face less dramatic temperature variations between summer and winter, like grassland rodents (Paise and Vieira, 2006; Pita et al., 2011). Photoperiod-dependent entrainment of the SCN is the very first step of photoperiod processing within the seasonal neuroendocrine physiology in mammals (see below). Thus, changes in endogenous activity α are not only a proxy for the SCN entrainment but may also indicate a preliminary photoperiodic response within the body.

In summary, our results support that tuco-tucos display seasonal differences in daily activity patterns, due both to endogenous and exogenous factors. The endogenous component is best shown by change in activity onset and offset times between summer and winter, indicating that tuco-tucos start activity time earlier and finish it later in summer, compared to winter. Within this endogenously regulated α interval, the animals emerge to the surface at random times, but the probability of exiting the tunnels increases with a season-dependent combination of subterranean and surface temperatures, which are the exogenous factors that shape their time on surface.

### Tuco-Tucos Are Able to Encode Photoperiod Information

The seasonal variations in tuco-tucos’ time on surface generate different light exposure patterns. Our next step was to verify if the contrasting parameters of light exposure between summer and winter could account for photoperiod measurement in tuco-tucos, via mathematical modeling. The α aftereffects of natural photoperiod (Figure 3) and splitting of activity/temperature rhythms into two bouts under LL (Figure 4) set the stage for a two-oscillator model of the circadian clock for photoperiod encoding in tuco-tucos. In addition, our light-logger data (Figure 1) provided the basic parameters used to simulate tuco-tucos’ natural daily light exposure patterns in summer and winter.

Several mechanisms have been proposed for photoperiodic time measurement and the circadian clock plays a central role in one such proposition, namely the “internal coincidence model.” It proposes that different photoperiods are transduced in the form of internal reorganization among components of the circadian system (Pittendrigh, 1972; Tackenberg and McMahon, 2018). In mammals, this model is supported by photoperiod-induced changes in the daily electrical activity of the SCN (VanderLeest et al., 2007), which is likely mediated by the phase relationships among component oscillators within the nucleus (Evans et al., 2013; Yoshikawa et al., 2017; Olde Engberink et al., 2020).

In its simplest form, the “internal coincidence” within the circadian clock can be modeled as the two oscillators E (evening) and M (morning), proposed by Pittendrigh and Daan (1976c). The E-M model was developed to explain circadian activity rhythms in nocturnal rodents, including the splitting of activity rhythms under constant light conditions (Pittendrigh and Daan, 1976c), which we also see in tuco-tucos (Figure 4; Valentinuzzi et al., 2009). In this model, as the day length varies throughout the seasons, there is an adjustment of the phase relationship between E and M (ΔΦ_*EM*_) (Figure 5). In our previous study, we used mathematical modeling to simulate this two-oscillator clock and how it transduces different photoperiods into changes in ΔΦ_*EM*_, provided that “dawn” and “dusk” signals input separately on M and E oscillators, respectively (Flôres and Oda, 2020). The model successfully replicated the correlations between photoperiod, ΔΦ_*EM*_ and activity α. The same model has also replicated the splitting phenomenon in constant conditions (Oda and Friesen, 2002).

The former E-M simulations (Flôres and Oda, 2020) were performed under “skeleton photoperiods,” a minimal representation of photoperiod in artificial lighting experiments (Pittendrigh and Daan, 1976b; Olde Engberink et al., 2020; Tackenberg et al., 2020). It consists in two light pulses per day, applied at the times corresponding to lights-on and off in complete photoperiod regimens (Figure 5). In the present study, the E-M model was exposed to light inputs that mimic the light exposure of tuco-tucos in the field. In contrast to skeleton photoperiods, in the new light regimens the pulse times were distributed randomly within the photophase, not at a fixed time. In principle, these regimens inspired by tuco-tucos’ light exposure should carry even less information about the timing and duration of the photophase, posing a greater challenge to photoperiod encoding.

Three light exposure models were tested, with increasing complexity, and we verified their capacity to modify ΔΦ_*EM*_ as a function of photoperiod. Surprisingly, photoperiodic information was already conveyed by the simplest light exposure model (Model I). Expectedly, the light inputs became more effective in informing photoperiod as we added further complexity in Models II and III. The additional features in these models more closely resemble the light/dark pattern experienced by tuco-tucos in the field. Nonetheless, the models are still gross approximations to the rich natural LD cycles, which also include changes in light intensity and spectral composition. The effectiveness of the simplified light exposure models, even in the absence of these natural features, corroborates that tuco-tucos get much more temporal information from their light exposure patterns than the theoretical minimum needed to decode photoperiods.

From a functional perspective, the ability to decode photoperiod is relevant not only to the adjustment of seasonal activity, but also as an anticipatory cue to time seasonal physiology in general. In mammals, the photoperiod-induced changes in the SCN trigger downstream seasonal physiological responses (Goldman, 2001). One crucial output of the SCN in this photoperiodic signaling cascade is the rhythm of melatonin secretion in the pineal gland. In nocturnal mammals, photoperiod modifies equally the durations of activity α and of nocturnal melatonin release (Illnerová and Vanìèek, 1982; Elliott and Tamarkin, 1994), as a result of photoperiod-dependent entrainment of the SCN. Sequentially, the duration of nocturnal melatonin informs photoperiod to downstream neuroendocrine systems that control seasonal reproduction (Dardente et al., 2019; Nakane and Yoshimura, 2019). In the tuco-tucos (*Ctenomys* spp.), most species studied so far display a seasonal reproductive pattern (Fanjul et al., 2021). Moreover, laboratory studies of female reproductive status under artificial photoperiods provided some evidence of photoperiodic responsiveness in *C. talarum* (Fanjul and Zenuto, 2008). Identifying these seasonal reproductive events, as well as other seasonal physiological changes, may provide clear output phase markers for future experiments, which would confirm neuroendocrine responses to changes in photoperiod.

Putting it all together, tuco-tucos display seasonal differences in time on surface, which results in seasonal differences in light exposure, transduced into different photoperiods. Conversely, the photoperiodic signals feed-back on the internal organization of the LD-entrained SCN, contributing to the different seasonal activity patterns. Understanding the complementary regulation of seasonal physiology by endogenous clocks and temperature in natural settings is urgent, in view of disruptions to phenology across the globe, associated with global warming (Walker et al., 2019). This knowledge is needed to estimate vulnerability of a species to the growing misalignment between photoperiod and seasonal environmental conditions caused by climate change (Walker et al., 2019; Meyer et al., 2021).

## Data Availability Statement

The raw data supporting the conclusions of this article will be made available by the authors, without undue reservation.

## Ethics Statement

Trapping and experimental procedures were approved in Argentina by the Environmental Secretary*—Ministerio de Planeamiento e Industria* (Expte. # P4 00501-17-2018)—of La Rioja, and by CICUAL (Institutional Committee for the Care and Use of Laboratory Animals) of *Facultad de Ciencias Veterinarias, Universidad de La Plata* (Protocol # 29-01-12), Buenos Aires province. They were also approved in Brazil by the institutional Animal Use Ethics Committee (CEUA) of the *Instituto de Biociências*, *Universidade de São Paulo* (Permits # 153/2012, 252/2016 and 273/2016).

## Author Contributions

GO and VV conceived the experiments. MJ, GI, PT, and VV performed experiments and analyzed data. MJ developed statistical modeling. DF and GO developed mathematical modeling. GO, DF, and MJ wrote the manuscript. All authors reviewed and approved the final manuscript.

## Conflict of Interest

The authors declare that the research was conducted in the absence of any commercial or financial relationships that could be construed as a potential conflict of interest.

## Publisher’s Note

All claims expressed in this article are solely those of the authors and do not necessarily represent those of their affiliated organizations, or those of the publisher, the editors and the reviewers. Any product that may be evaluated in this article, or claim that may be made by its manufacturer, is not guaranteed or endorsed by the publisher.
