# Supplementary material for: Telling the Seasons Underground: The Circadian Clock and Ambient Temperature Shape Light Exposure and Photoperiodism in a Subterranean Rodent
Source: Front Physiol. 2021 Oct 1;12:738471. doi: 10.3389/fphys.2021.738471 (PMC8517108; doi:10.3389/fphys.2021.738471)
Supplement: Supplementary file 1 [file Data_Sheet_1.pdf]

Supplementary Material

1 Supplementary Text

Random light pulse time generation and selection

Random pulse times for the mathematical model were generated in R. For each simulation in Model I, we produced 1 batch of 50 pulse times, to cover the 50 days of light pulses (1 pulse per day, Figure 6B, Supplementary Figure 4). For Models II and III we generated 2 batches of 50 pulse times, one for each of the 2 daily intervals of pulse distribution (2 pulses per day, Figures 6C-D, Supplementary Figure 4). Within each pulse batch, the times were generated as random numbers drawn from a uniform distribution within the pre-defined time interval.

Each batch of 50 pulses was chosen from a sample of 4000 batches generated simultaneously. The following selection criteria were used to choose the batch: a) uniformity of the distribution of the pulse times within the pulse time window and b) spread of the pulse times in consecutive days. Pulse time uniformity within the time window was quantified with the Kolmogorov-Smirnov (KS) test. Pulse time spread in consecutive days was evaluated in a spatial plane of day versus time. The spatial spread of the pulses times in the plane was quantified with the Clark-Evans (CE) test. We selected the batch with the lowest KS D statistics and the highest CE R statistics within the 4000 batches. To that end, the D and R statistics were each transformed to a 0 to 1 scale, with 0 as the lowest value observed within the 4000 batches and 1 as the highest value within the batches. Then, we chose the batch with the shortest Euclidian distance to the point (0,1) in the D versus R plane.

2 Supplementary Figures and Tables

2.1 Supplementary Figures 1-5

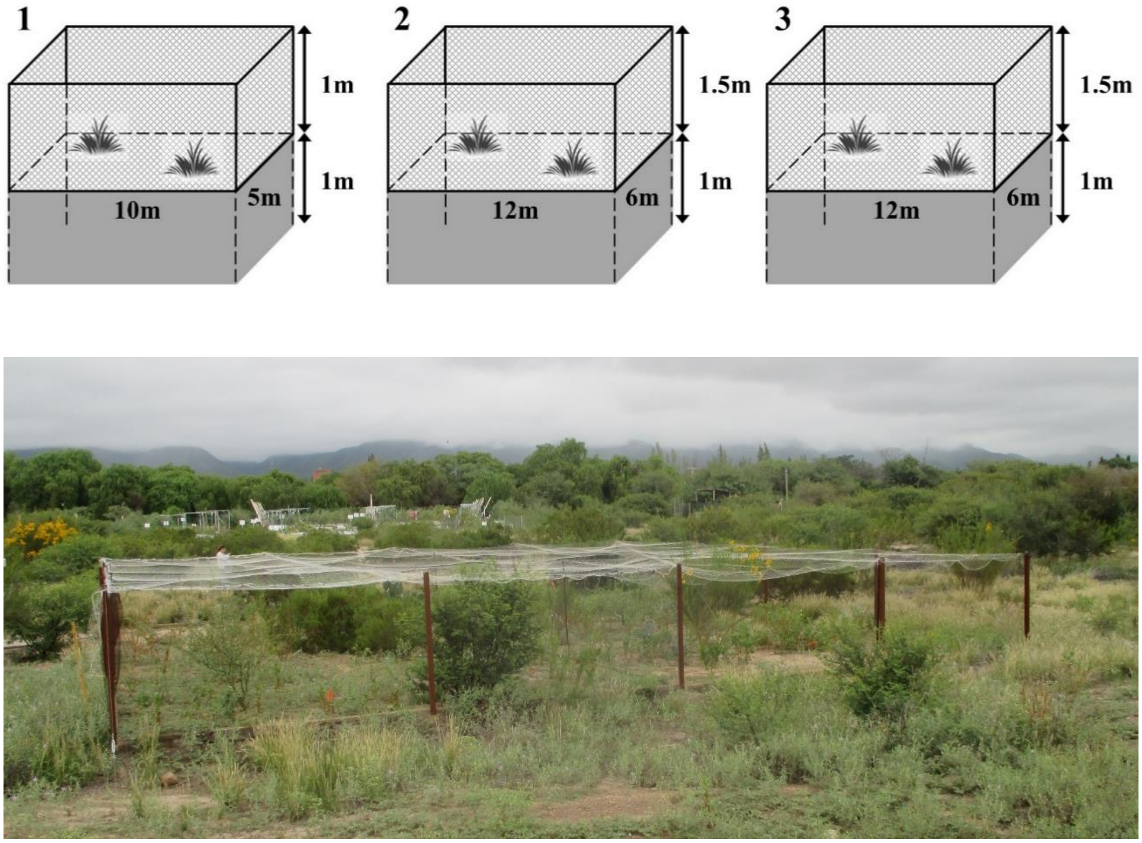

Supplementary Figure 1. Overview of semi-natural enclosures built inside CRILAR, Anillaco, La Rioja, Argentina. Top: schematic figures describe each enclosure’s dimensions above (light gray) and below ground (dark grey). All of them are delimited underground by concrete walls. Bottom: a photograph of enclosures 2 and 3, built next to each other.

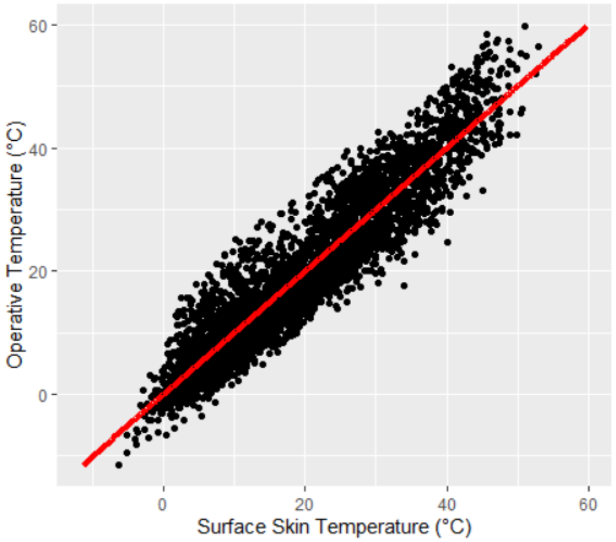

Supplementary Figure 2. Fitting of measured values to the prediction of the linear model of operative temperature (T_e) as a function of surface skin temperature (T_{skin}). Measures (black points) of T_e and T_{skin} follow a positive linear relationship dependent on season (not shown, for simplicity) and this relationship is predicted by the model (red line) with an R^2 of 87.44% and $p<0.001$.

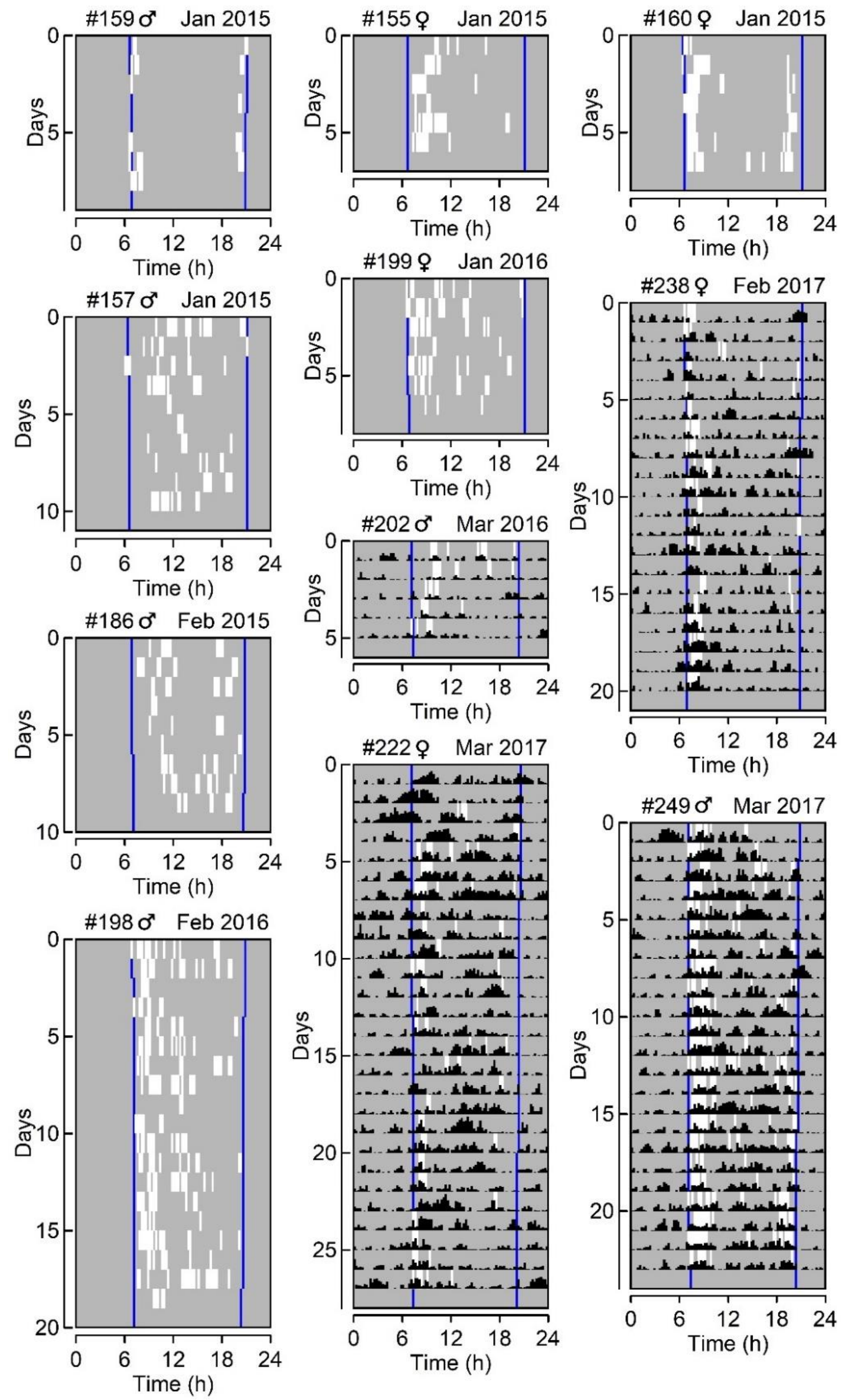

Supplementary Figure 3. Short term recordings of light exposure (white) and general activity (black) of all recaptured animals from semi-natural enclosures during summer seasons of 2015, 2016 and 2017. Seven animals had only light loggers while four of them had both light and activity loggers. Blue lines correspond to civil twilight limits.

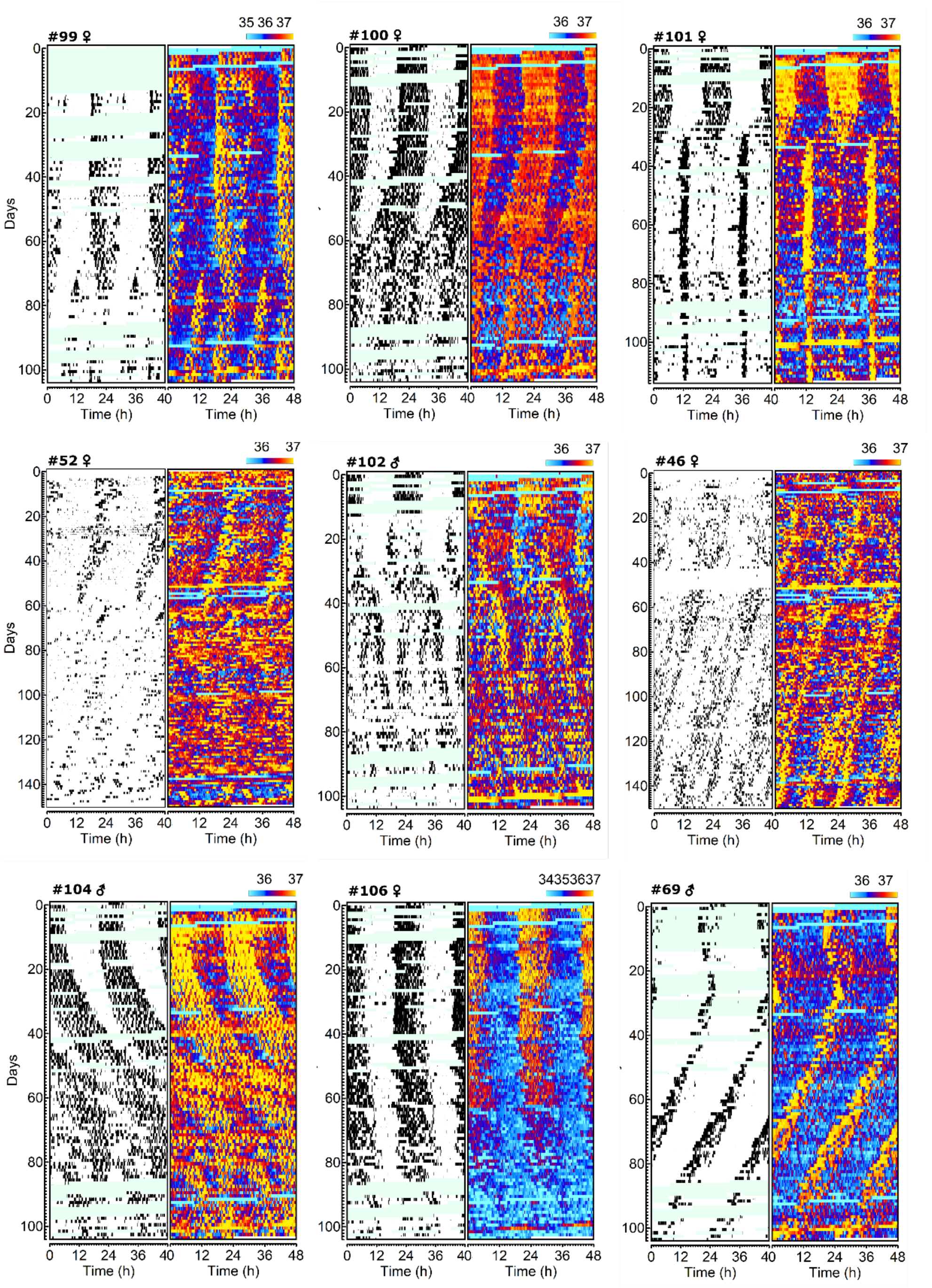

Supplementary Figure 4. Wheel running and body temperature rhythms of tuco-tucos under prolonged constant light. Of the nine animals submitted to the constant light protocol, seven (Animals #46, #52, #99, #100, #101, #102 and #104) showed splitting events while two (Animals #69 and #106) did not. Left of each panel: double-plotted actogram depicts wheel running (black bars indicate when running was recorded). Right of each panel: body temperature actogram (a color gradient is used to display temperature values). Areas shaded in light green indicate missing data. Constant light (LL) started on day 14, preceded by LD cycle (12:12, lights on at 07:00).

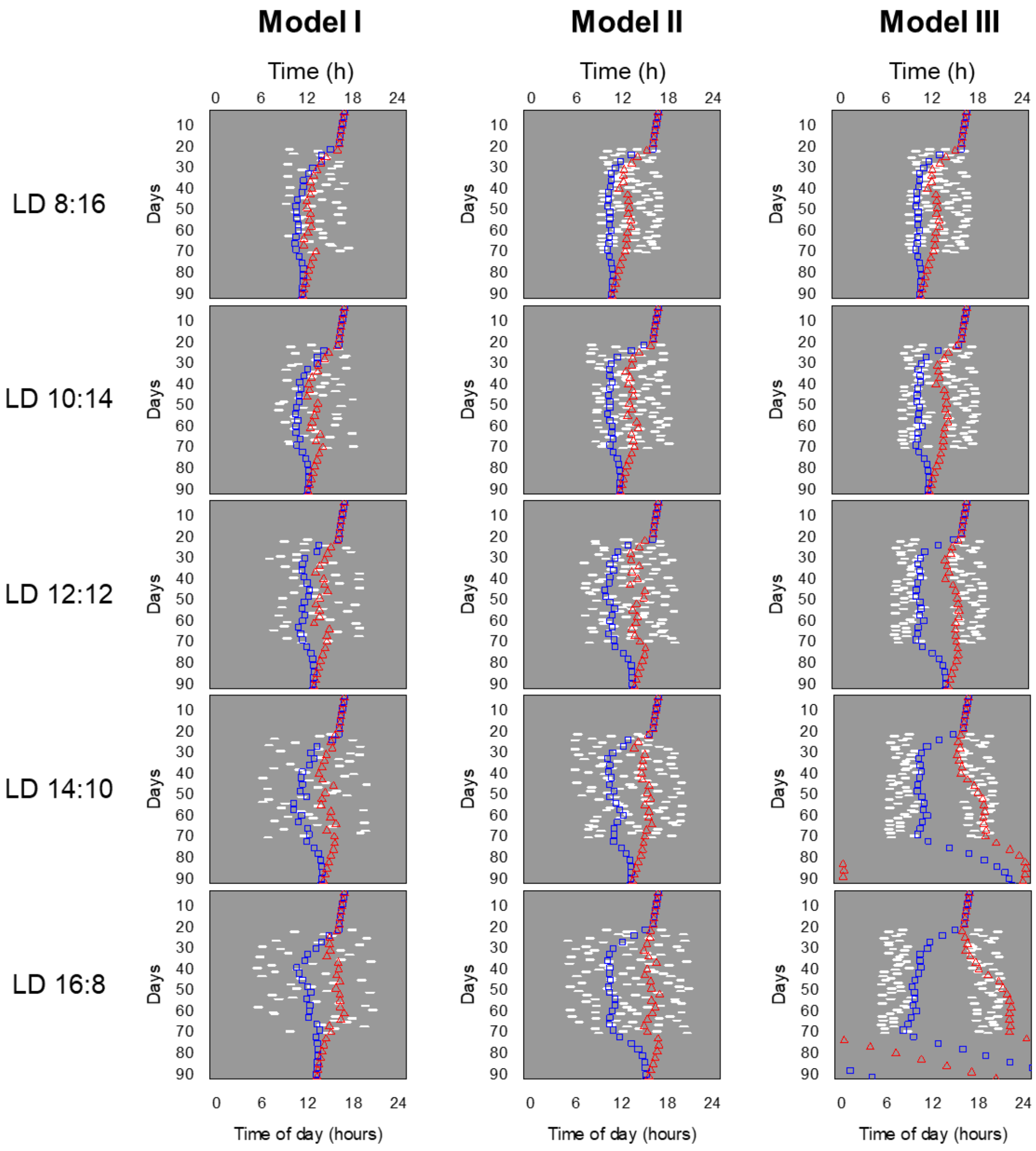

Supplementary Figure 5. Dynamics of the morning-evening oscillator model under different photoperiods, in the 3 light exposure models. Morning (M) and evening (E) coupled oscillators were exposed to 20 days of constant darkness, followed by 50 days under the light pulses (white marks) and another 20+ days in constant darkness. In each actogram, colored symbols depict the reference phases of M (blue squares) and E (red triangles), plotted only every third day for better visualization. See main text for details on the light exposure models. Other model parameters are described in the Materials and Methods.
